# Constitutions and Temperaments

**Published:** 1851-03

**Authors:** Silas Hubbard

**Affiliations:** Buffalo


*Constitutions and Temperaments.* By SILAS HUBBARD, M. D.

TO THE EDITOR OF THE BUFFALO MEDICAL JOURNAL:

SIR,—Having read a paper before the Erie County Medical Society at its last semi annual meeting, which received the favorable attention of the society, I am induced to hope that it may possess some interest to those of your readers who did not hear it, and that it may not appear entirely presumptuous in me to offer the same for publication. I therefore transmit to you the substance thereof, as follows:

GENTLEMEN:—On this occasion, I choose for the subjects of my discourse, the Constitutions and Temperaments. It will be perceived that in employing the words constitutions and temperaments, I make a distinction between them. Writers, both ancient and modern, have, in their imperfect description of the temperaments, confounded with them the constitutions. Without now stopping to show the particular defects of either ancient or modern writers, I shall proceed directly to give a very brief digest of the subjects of my discourse.

The constitutions, as I shall now define them, are those natural causes of the differences of individuals, indicated by the developements of the head, thorax, abdomen, and the osseous and muscular systems.

The *temperaments* are those natural causes of the differences of individuals, produced by the qualities of the *constitutions*, and indicated by the complexion of the hair, eyes, and skin, texture of the muscular system, &c.

The constitution, as a whole, is divided into four principal classes, or heads, as already mentioned, which I shall name as follows: 1st, the Cerebral; 2d, the Thoracic; 3d, the Digestive; 4th, the Motive;—and shall treat of them in that order.

I. *Cerebral.* The size of this constitution is generally indicated by the developement or extent of the external periphery of the cranium. The brain, as a whole, is the organ of all those intellectual faculties and affective feelings, of which we can become conscious in this life. It is intimately connected with every portion of the economy, through the medium of the nervous system, and is related to every phenomenon of animal life. I shall not here give the anatomy, or discuss the physiology of the brain; but if we regard the principles of phrenology to be true, it will be seen that the peculiarities of developement, or structure of the brain, must constitute a great source of differences of individuals: but whether the details of phrenology are true, or not true, still the brain, by reason of its predominance or inferiority, has the power of modifying the whole economy—probably to a greater extent than any other portion of the general constitution.

On this occasion, I would scarcely be able to give more than a plan of the tendencies of the palpable external developements of this constitution. For this reason, I shall merely recommend to every physician the particular importance of studying the size and external conformations of the cranium as far as he can, in indicating the physical, moral, and intellectual peculiarities of individuals—both in children and adults.

II. *Thoracic.* The thorax is one of the splanchnic cavities; bounded, posteriorly, by the vertebræ; laterally, by the ribs and scapula; anteriorly, by the sternum; above, by the clavicle; and below, by the diaphragm. It is destined to lodge and protect the chief organs of respiration and circulation—the lungs and the heart. "The lungs and heart, as a whole, have for their principal functions the arterialization and circulation of the blood; and also largely participate with other vital organs in the production of calorification; and are thus the principal vivifying organs of the whole system. The capacity and power of these organs, and their room for action, can generally be indicated during life by the external dimensions of the chest, and width of the shoulders. The capacity for forcible respiration, and power for exercise, are generally greatest at about thirty years of age. The general health and power of the individual, and the capacity for exercise, are dependent to a great degree on the developement of this constitution. Its external developement can, (as can indeed the developement of each constitution,) be made an indication of predisposition to health and disease to a considerable extent; but which I do not now propose particularly to consider.

III. *Digestive.* By this term I particularly mean the abdominal viscera. The abdomen is the largest of the three splanchnic cavities; bounded, above, by the diaphragm; below, by the pelvis; behind, by the lumbar vertebræ; and at the sides and fore part, by muscular expansions. The chief viscera contained in the abdomen are the stomach, intestines, liver, spleen, pancreas, kidneys, &c.

There is considerable difference of capacity of the abdomen in different individuals, which will generally be developed in proportion to the size, or capacity of the contained organs. There are even national differences in this respect. The Germans and English are greater eaters than the French or Spaniards. Some people, especially after the middle age, have a tendency to corpulency, and possess a great developement of this cavity, which is not supposed to indicate particular size or activity of the digestive organs. It is not important that I should now fully state the causes of this developement; but suffice it to say, that such persons are generally good livers, or particularly fond of good living. A full developement, and good degree of activity of the digestive organs are favorable; but there may be an excessive, or an insufficient developement of these organs.

IV. *Motive.* I call this the motive constitution, because it comprises the osseous and muscular systems—the principal mechanism for all muscular movements. The osseous and muscular substances are generally duly proportioned to each other. They generally constitute the principal bulk of the system as a whole. The developement of this constitution causes the principal difference of the size of individuals. Men have a larger endowment of this constitution than women. The peculiar and relative state of organization, or developement of this, and the cerebral constitution—being the chief mechanism for all muscular movements and mental operations—principally decide the proper avocations of individuals.

I have thus briefly and imperfectly described the principal constitutions, more for the purpose of giving form to my plan of drawing a line of distinction between them and the temperaments, than the expectation of throwing extensive light on either. The constitutions are intimately connected with, and related to each other. I shall not attempt even to name all these relations, but will briefly notice a few of those most palpable. The organs of circulation are intimately connected with, and dependent on the digestive organs; in the formation of blood; and every portion of the human system is dependent on the circulatory and respiratory organs for nutrition, calorification, vivification, &c. Also, every portion of the whole system is dependent on the cerebral constitution as the principal organ of innervation. Some of these relations existing between the encephalic organs and other portions of the constitution, I shall briefly allude to, on account of their supposed external palpable nature. Dr. Hoppe professes to have discovered the cerebral organ of Alimentiveness, appetite for sustenance, situated in the anterior inferior portion of the middle lobe of the cerebrum. Mr. J. S. Grimes professes to have discovered the organ of Pneumativeness, presiding over the respiration, located contiguous to Alimentiveness, and that there is a relation existing between the developement of the head indicating the size of Alimentiveness, and the digestive organs; also, that there is a relation existing between the developement of the head indicating the size of Pneumativeness, and the respiratory organs. Some learned and zealous opponents of phrenology, in their arguments against it, admit that there is a corresponding relation existing between the developements of the head, mapped by phrenologists as Alimentiveness, and the digestive organs; and of Pneumativeness, and the respiratory organs; and that the strength of the propensities in question are in proportion to the respective development of said organs: but they account for this apparent mutual relation of developement of parts and power on very different principles from those of the phrenologists. I shall not here assume the province of deciding who are the nearest correct; both may be correct to a certain degree; but the fact that there is a correlation existing between the developements of the portions of the head in question, and the principal digestive and respiratory organs is worthy of record.

Some phrenologists, and also anti-phrenologists, are agreed that broad heads are generally associated with strength and vigor of constitution, or athletic powers. It seems to me that there is a greater coincidence between the developement of the cranium indicating the size of the cerebellum, and the athletic powers or motive constitution, than any other portion of the cranium. So striking is this coincidence of developement, that I am inclined to believe what several authors have hinted, namely, that the cerebellum is an animal organ which "has considerable reference to the strength and perfection of the bodily frame." Indeed I would advance the opinion that its chief function is to preside over *nutrition*. In proof of this observation, I might adduce the following facts—1st, the cerebellum attains its fullest size at the time the bodily frame acquires its fullest perfection; 2d, not only is this difference of size of the cerebellum, and developement of the athletic powers, observable in individuals of the same sex, but—3d, the cerebellum and osseous and muscular system are generally larger developed in the male than in the female. 4th, not only is this true of man, but of the animal creation generally, which possess the cerebellum, and—5th, the lower the animal in the scale of organization, the more deficient is the cerebellum. 6th, I have observed that those possessing the cerebellum small are more subject to perversions of nutrition than those with it large or full. 7th, I have observed that where the cerebellum is small or moderate, or the cerebrum much predominates in size over the cerebellum, the individual is possessed of a delicate constitution, and is likely to belong to that class described by authors as of the nervous temperament. I do not wish to be understood that it is my opinion, that it is the sole function of the cerebellum to preside over nutrition, or the developement and maintenance of the bodily frame—it may have other functions; but I have not wished to speak of it only so far as it seems to relate to the subjects in question.

Having thus described, though imperfectly, the principal constitutions, I shall now proceed briefly to notice

*The Temperaments.*—The temperaments, as previously defined, are those natural causes of individual differences, produced by the qualities of the constitutions, and indicated by the complexion of the hair, eyes, and skin, texture of the muscular system, &c. In this discourse I shall only treat of them as they are exhibited in the Caucasian race. And before giving my own views upon them, I shall briefly state the manner in which they have been treated of by ancient and various modern authors; and shall then adopt such portions of their opinions, and make such criticisms and substitutions as I think most useful. The ancients generally admitted four, denominated from the respective fluids or humors, the superabundance of which in the economy was supposed to produce them; the *sanguineous*, caused by a surplus of blood; the *bilious* or *choleric*, produced by a surplus of yellow bile; the *phlegmatic*, caused by a surplus of phlegm, lymph, or fine watery fluid derived from the brain; and the *atra-biliary* or *melancholic*, produced by a surplus of black bile—the supposed secretion of the atra-biliary capsules and spleen.

This division was kept up for ages, without modification, and still prevails with one or more additional genera. The epithets have been retained in popular language without our being aware of their parentage. For example, we speak of a *sanguine*, *choleric*, *phlegmatic*, or *melancholic* individual or turn of mind, with nearly the acceptation given to them by the Hippocratic school—the possessors of these temperaments being presumed to be, respectively, full of high hope and buoyancy; naturally irascible, dull and sluggish; or gloomy and low-spirited. Metzger admits only two—the *irritable*, (*reizbare*,) and the dull or *phlegmatic*, (*trage*.) Urisberg, eight—the *sanguine*, *sanguineo-choleric*, *choleric*, *hypochondriac*, *melancholic*, *Bœotian*, *meek*, (*sanftmuthige*,) and the *dull* or *phlegmatic*. Rudolphi, also, eight—the *strong* or *normal;* the *rude; athletic* or *Bœotian;* the *lively;* the *restless;* the *meek;* the *phlegmatic* or *dull;* the *timorous*, and the *melancholic;* while Broussais enumerates the *gastric*, *bilious*, *sanguine*, *lymphatico-sanguineous*, *anœmic*, *nervous*, *bilioso-sanguine*, *nervoso-sanguine*, and *melancholic*. It is obvious, that if we were to apply an epithet to the possible modifications caused by every apparatus of organs, the number might be extended much beyond any of these. If we should regard phrenology as true, then just from the state of developement of the cerebral constitution alone, we might multiply the temperaments to a greater extent, and with as much propriety as many of those above named. We might enumerate as many as there are different mental and moral qualities of individuals, or in other words as there are organs of the brain, or as many as we may choose from combinations of organs. "Perhaps the division most generally adopted is that embraced by Richerand, who has embodied considerable animation, with much that is fanciful, in his description." These temperaments are the *sanguine*, the *bilious*, or *choleric*, the *melancholic*, the *phlegmatic*, and the *nervous*. In giving my description of the temperaments, I shall principally make requisitions on this last division.

The temperaments, as I shall divide them, are three:—1st, the *Nervo-sanguine* or *active temperament*; 2d, the *Bilious* or *enduring temperament*; 3d, the *phlegmatic* or *easy temperament*.

I.—*The Nervo-sanguine* or *Active Temperament*. This temperament is indicated by sandy, or chestnut, or light colored hair; florid complexion, or fair skin; light, or blue, or gray eyes, and firm flesh. If the general constitution is well developed, it is accompanied with strong, frequent, and regular pulse. The particular qualities of this temperament are, superior nervous susceptibility, and excitability and activity of mind and body.

Every thing else being equal, all the functions partake of more activity, than in the other temperaments. The peculiar complexions and characteristics of this temperament, I suppose are produced by a predominance of arterial blood, or by some inappreciable modification of the nervous and circulatory systems. This temperament has frequently been confounded with a predominant cerebral constitution, or an impaired or diseased constitution, and called the nervous temperament; or with a predominant thoracic constitution, with a moderate cerebral constitution, &c., and called the sanguine temperament. Every other circumstance being equal, the diseases of this temperament, are generally more violent, and oftener seated in the circulatory and nervous systems; as fevers, inflammations, hemorrhages, and nervous complaints, than in the other temperaments.

II.—*The Bilious* or *Enduring Temperament.* This temperament is indicated by dark hair, dark eyes, and dark or brown skin, or skin inclining to yellow, muscles firm, subcutaneous veins prominent. Every other condition being equal, the pulse is rather stronger and harder, though not quite so frequent as in the *nervo-sanguime* or *active temperament.* Individuals of this temperament, *cœteris paribus,* are stronger, and capable of enduring protracted mental, or physical fatigue or exercise, better than those of the other temperaments; they are not so often precocious, or susceptible to the effects of medicine, or external influences, as those of the preceding temperament. The diseases are generally combined with more or less derangement of the hepatic system. We are not entirely certain what are the causes of the peculiar complexions, and qualities of this temperament; they may be produced by a peculiar structural organization of the liver, or biliary organs in general, or by a predominance of dark or venous blood, or it may be both causes combined. Richerard enumerates as examples of what he calls the bilious temperament, Alexander, Julius Cæsar, Brutus, Mahomet, Charles XII, Peter the Great, Cromwell, Sextus V, and the Cardinal Richelieu. To these, Good has added Attila, Charlemagne, Tamerlane, Richard III, Nadir shah and Napoleon. Richerard and Good do not mention the fact, that these great men derived their superior abilities, as much from a favorable endowment of the constitutions, as from the peculiar qualities imparted by this temperament. This temperament has been combined by various authors with a small, or moderate endowment of the thoracic consiitution, or with derangement of some of the abdominal organs, or in the nervous system, and called the melancholic or atrabilious temperament.

III.—*The Phlegmatic* or *Easy Temperament.* This temperament, I

think, is better defined by authors, than any of the others. The following by Dunglison, is the usual description of this temperament; which he calls the *Phlegmatic, Lymphatic* or *Pituitous Temperament.*—"In this case, the proportion of the fluids is conceived to be too great for that of the solids; the secretory system appearing to be active, whilst the absorbent system does not act so energetically as to prevent the cellular texture from being filled with humors. The characteristics of this temperament are:—soft flesh; pale skin; fair hair; weak, slow, and soft pulse; figure rounded, but inexpressive; the vital actions more or less languid; the memory by no means tenacious, and the attention vaccillating; with aversion to both mental and corporeal exertion. Pomsonious Atticus, the friend of Cicero, is offered as an example of this temperament, in ancient times; Montaigne, in more recent history. The latter, however, possessed much of the nervous susceptibility that characterizes the more lively temperaments. Dr. Good suggests the Emperor Theodosius as an example in earlier times; and Charles IV of Spain, who resigned himself almost wholly into the hands of Godoy,—Augustus, King of Saxony, who equally resigned himself into the hands of Napoleon,—and Ferdinand of Sicily, who surrendered for a time the government of his people to the British,—as instances in our own day. It would not be difficult to find, amongst the crowned heads of Europe, others that are equally entitled to be placed amongst these worthies." This temperament, I think, has suffered too much disparagement in comparison with the others; so much so, that no individual would think himself at all honored to be called of the phlegmatic temperament. I call it the *easy temperament*, because the repletion of the cellular tissue, and other peculiarities of it, disposes the individual to quietude, and thus protects all of the organs from injury by too powerful exercise of their functions, and by the defence afforded by the secretions. Every person possesses more or less of the qualities which constitute this temperament; and which are essential, to a certain degree, to our very existence: but we rarely speak of this temperament, excepting when these qualifying causes exist so superabundantly, as to be quite palpable, and to almost constitute morbid conditions.

Such are the temperaments as they seem to me. There may be other qualities of all the constitutions, produced by fineness, or coarseness, or intimacy of structure, which cannot be detected during life, only by their effects; some of them may be seen by dissection, but which I shall not dwell upon in this discourse, which may have been already protracted too long for your patient hearing. I shall also avoid further protracting this discourse, by not even naming trifling points of differences, and by omitting to speak particularly of the constitutions and temperaments, as modified by parentage, age, sex, national differences, habit, cultivation, climate, food, disease, &c.

I shall conclude this discourse by recommending to you the great importance of studying the constitutions and temperaments as they are developed by external indications. They are sufficient of themselves to constitute a large volume. It would be useful if physicians would note the constitution and temperament of all their patients, and ascertain all the diseases to which they have been subjected. We might thus form statistics of all the diseases most likely to be associated with the various constitutions and temperaments. Not only might we make a proper understanding of the constitutions and temperaments a source of immense advantage in understanding the causes and proper treatment of diseases: but it may also be made a source of immense advantage, in directing the moral, intellectual, and physical culture of mankind.

BUFFALO, January, 1851.

